# Cardioneuroablation Using Epicardial Pulsed Field Ablation for the Treatment of Atrial Fibrillation

**DOI:** 10.3390/jcdd10060238

**Published:** 2023-05-29

**Authors:** Barry O’Brien, John Reilly, Ken Coffey, Ana González-Suárez, Leo Quinlan, Martin van Zyl

**Affiliations:** 1 AtriAN Medical Ltd., Unit 204, Business Innovation Centre, Upper Newcastle, H91 W60E Galway, Ireland; 2 School of Engineering, University of Galway, H91 TK33 Galway, Ireland; 3 Translational Medical Device Lab, University of Galway, H91 YR71 Galway, Ireland; 4 Physiology and Cellular Physiology Research Laboratory, CURAM SFI Centre for Research in Medical Device, University of Galway, H91 TK33 Galway, Ireland; 5 Cardiac Electrophysiology, Royal Jubilee Hospital, Victoria, BC V8R 1J8, Canada

**Keywords:** atrial fibrillation, ganglionated plexi, cardiac autonomics, cardioneuroablation, pulsed electric field

## Abstract

Atrial fibrillation (AF) is the most common cardiac arrhythmia affecting millions of people worldwide. The cardiac autonomic nervous system (ANS) is widely recognized as playing a key role in both the initiation and propagation of AF. This paper reviews the background and development of a unique cardioneuroablation technique for the modulation of the cardiac ANS as a potential treatment for AF. The treatment uses pulsed electric field energy to selectively electroporate ANS structures on the epicardial surface of the heart. Insights from in vitro studies and electric field models are presented as well as data from both pre-clinical and early clinical studies.

## 1. Introduction

Atrial fibrillation (AF) is the most common cardiac arrhythmia with an increasing incidence rate, even when the figures are adjusted for population aging [1]. It is estimated that up to 16 million people in the US will have AF by 2050, 14 million in Europe by 2060, and over 70 million in Asia by 2050 [2]. AF increases the risk of stroke by a factor of 3–5, and it is estimated that AF is responsible for 15% of all strokes worldwide [3]. The risk of developing heart failure is increased five-fold in patients with AF [4]. Despite this growing epidemic, treatments continue to be suboptimal. Anti-arrhythmic drugs (AADs) are usually prescribed first as they are considered the least invasive first line of treatment. Mostly, AADs have a success rate of only 30–35% at 1-year follow-ups [5]. Amiodarone is one of the most effective AADs, with up to 65% of patients in sinus rhythm at 1-year follow-up; however, long-term use of this drug is limited by its cumulative dose-dependent toxicity to many organs, including the lungs, thyroid and liver [6]. 

Catheter ablation is the next option for patients in whom the AADs fail or when the side-effects can no longer be tolerated. Ablation techniques aim to electrically isolate the left atrium from the pulmonary veins by creating circumferential lesions around the vein ostia or antral region. This approach was built on the observation that pulmonary veins were the major source of triggers for fibrillation [7]. Radiofrequency (RF) heating was the ablation technology of choice for many years, with the use of low-temperature cryoballoons being subsequently introduced. From an efficacy perspective, both techniques give similar outcomes as demonstrated in the FIRE AND ICE trial, where a 1-year success rate of approximately 65% was achieved in patients with paroxysmal AF [8]; however, this relatively modest success rate typically drops off steadily over time. For example, the FreezeAF study showed that the single-procedure success rate was only 40% and 42% for RF and cryoballoon, respectively, for paroxysmal AF, at 30-months follow-up [9]. Repeat ablation can be used to improve on these results. Outcomes for persistent and permanent AF are typically worse than paroxysmal. While recent technology iterations, such as second generation cryoballoons and contact-force RF catheters, have come to market, they have not markedly improved efficacy outcomes [10,11].

## 2. Pulsed Field Ablation

Direct current (DC) ablation—the predecessor to modern pulsed field ablation (PFA)—was originally explored in the 1980s for treatment of cardiac arrhythmias; however, complications such as coronary sinus injury [12] and proarrhythmic effects [13] were reported in early studies. These issues were typically due to technology limitations, with too much energy being delivered per treatment pulse. Specifically, the high voltages and long pulse durations were enough to cause arcing in the aqueous blood environment with the rapid creation and collapse of gas bubbles; the resulting high-pressure shock waves being enough to cause vessel rupture, i.e., barotrauma [14]. Equipment development efforts subsequently focused on delivering the energy through trains of shorter pulses and determining pulse width, amplitude and intervals that could avoid arcing, but still deliver the energy desired for tissue ablation. Concurrent developments in RF ablation at the time surpassed these DC ablation techniques, and the approach lay dormant for many years until a more recent resurgence of interest, in part driven by successes in the oncology field [15] but also by a desire to improve on RF and cryoballoon outcomes from both an efficacy and safety perspective.

While the group at the University Medical Center Utrecht must be credited with some of the key pre-clinical research [16,17,18,19], the first prospective clinical trial data using PFA for treatment of paroxysmal AF was obtained using novel epicardial and endocardial devices developed by FaraPulse Inc. (Menlo Park, CA, USA), (since acquired by Boston Scientific, Marlborough, MA, USA) [20]. Several more devices are now in development [21], and trials have also extended to persistent AF patients [22]. The most encouraging aspect of all of these devices and trials has been the improved safety profile compared to RF and cryoablation tools. These conventional thermal approaches have been hampered by serious risks, such as atrioesophageal fistula [23], phrenic nerve damage [24] and pulmonary vein stenosis [25]. Pre-clinical work with PFA has shown that the oesophagus [26,27] and phrenic nerve [28,29] are spared from injury using energy parameters known to create myocardial ablation. This has since been supported by several thorough clinical investigations into these aspects [30,31]. Furthermore, deliberate PFA delivery inside the pulmonary vein does not appear to induce the same fibrotic reaction, which results in pulmonary vein stenosis as a result of conventional thermal ablation energies [32]. 

From an efficacy perspective, early indicators have been very promising as most studies are demonstrating near-100% electrical isolation of the veins acutely, as well as data from 3-month remapping showing a high rate of isolation persisting [33,34]. The 1-year follow-up data from the initial clinical trials have also been impressive with freedom from atrial arrhythmia of up to 84.5% reported [35]. Some of the first ‘real world’ data for this system is being gathered in the MANIFEST registry [36]; while these data further support the excellent safety profile of the treatment, the efficacy outcomes are not as high as those obtained in the clinical trial settings. Freedom from atrial arrhythmia at 1-year is reported at 81% and 71% for paroxysmal and persistent AF patients, respectively [37]. In this regard the results are marginally better than outcomes for RF and cryoballoon technologies. Considering the high rates of acute isolation achieved, it suggests that other physiological mechanisms, such as non-pulmonary vein triggers and residual atrial substrate, may be playing a significant role in AF.

## 3. Cardiac Autonomic Nervous System

**Figure 1:**
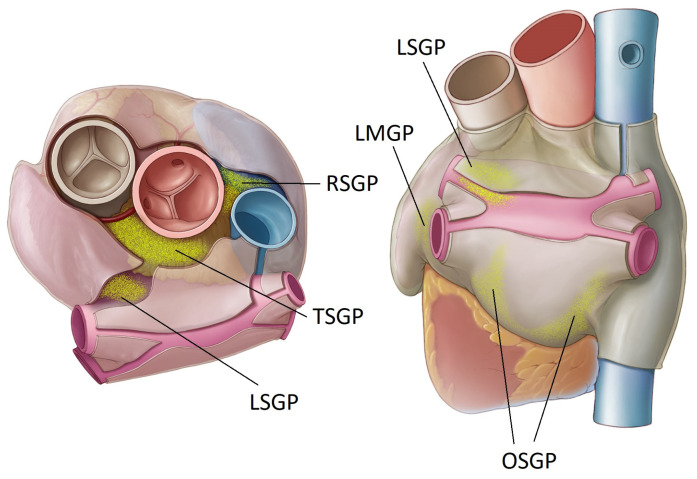
Location of key atrial ganglionated plexi (GP). Oblique sinus (OS), right superior (RS), transverse sinus (TS), left superior (LS) and ligament of Marshall (LM).

Autonomic innervation of the heart has long been recognized, though not necessarily fully appreciated, in the context of cardiac arrhythmias. Early animal studies by Armour demonstrated how atrial arrhythmia could be induced when these nerves were stimulated [38], while his subsequent work elucidated the precise anatomy and structure of this cardiac autonomic nervous system (ANS) in humans [39]. The heart is innervated by both the extrinsic and intrinsic cardiac ANS. The extrinsic consist of the ganglia in the brain and spinal cord (where the cell bodies reside) and the axons extending beyond this to the heart. The intrinsic cardiac ANS comprises an extensive neural network with clusters of ganglia interconnected by epicardial axons on the heart—these clusters are known as ganglionated plexi (GPs). The GPs are typically embedded in epicardial fat and can contain several thousand neurons, with both sympathetic and parasympathetic inputs, though the parasympathetic is considered to predominate. They are located primarily on the left and right atrium—mostly on the posterior surfaces—though there are also some ventricular GPs. There is an abundance of different nomenclature used to define GP locations. In this review paper we will use the system most closely aligned with an electrophysiologists perspective, as schematically shown in Figure 1.

- The right superior GP (RSGP) is located on the anterosuperior surface of the right atrium, medial to the superior vena cava/right atrium junction and lateral to the aortic root. It is also sometimes known as the aortocaval (SVC-Ao) GP.
- The left superior GP (LSGP) is located at the junction of the left superior pulmonary vein with the posterior left atrium, in the left superolateral area.
- The inferior right and left GPs (IRGP, ILGP) are located at the inferior aspect of the posterior wall of the left atrium, below the right and left PVs. These are sometimes identified as the oblique sinus GP (OSGP).
- The ligament of Marshall also contains autonomic neurons and is considered a target ganglion in this context (LMGP). This is located between the anterior aspect of the left pulmonary veins and the posterior left atrial appendage. It is also known as the vein of Marshall GP (VMGP).

The GPs influence a variety of cardiac physiological events, including sinus rate, AV node conduction and tissue refractoriness—innervation of the sinus node and AV node being most important. Credit must go to the research group at the University of Oklahoma for their early and on-going pre-clinical research that identified the key role that the GPs play in both initiating and sustaining atrial arrhythmias. AF induced by PV firing was shown to be controlled by stimulation of the GPs adjacent to the PVs—direct stimulation of the veins was not enough to induce AF [40]. Subsequently it was established that the heightened parasympathetic tone induced by stimulation of the GPs could result in atrial myocardial action potential shortening, shortened atrial effective refractory periods, and calcium-induced triggered atrial activity, thereby enhancing the substrate as well as the triggers for AF [40,41,42,43]. These pre-clinical observations were supported by early clinical work from Pappone et al. that indicated how the efficacy of PVI was higher when the ablation path intersected these retro-atrial GPs [44]. One of the earliest studies intentionally targeting the GPs for ablation was performed in Oklahoma. The GPs were identified by endocardial high frequency stimulation and ablated with RF—the addition of this step to the conventional PVI significantly reduced AF recurrence [45].

## 4. Ablation of Ganglionated Plexi—Early Studies

The first randomized trial assessing the benefit of adding GP ablation to PVI as well as assessing GP ablation alone was performed using endocardial RF ablation, with the GP sites identified anatomically [46]. At the two-year follow-up, freedom from AF was 74% for PVI + GP, 56% for PVI alone, and 48% for GP alone. The authors noted that conventional PVI probably intersects with a number of GPs and with other ganglia and nerves, i.e., PVI is already giving a partial autonomic denervation, but specifically targeting the GPs makes this more effective. It was noticed, however, that GP ablation increased the risk of left atrial tachycardia or flutter, most likely due to excessive ablation of healthy myocardium in the vicinity of the GPs. This effect of additional myocardial damage leading to tachycardia was also observed in a thoracoscopic GP ablation study [47]; this randomized study compared PVI against PVI with GP ablation (all epicardial and thoracoscopic), but high rates of tachycardia and flutter in the GP group was attributed to the additional myocardium damage that occurred during GP ablation. Despite mixed outcomes in these trials a meta-analysis of these and other GP studies still showed an added benefit of GP ablation compared to PVI alone, particularly for patients with paroxysmal AF [48]. Even in the context of re-treating patients with failed PVI (recurrent AF), an interesting study demonstrated that a further treatment of PVI combined with GP ablation gave better outcomes than patients treated with a repeat PVI alone. At 1-year follow-up, 90.6% of the repeat PVI with GP group were in sinus rhythm compared to 78% of the repeat PVI alone group [49].

An approach that could selectively ablate the GPs without damaging the myocardium would seem to be ideal in terms of properly discerning the potential anti-arrhythmic effect of GP ablation. Though not specifically aiming for permanent ablation of the GPs, an approach that involved injection of botulinum toxin into the fat pads/ganglia is highly selective [50]. This treatment was done in an open-chest surgery setting on patients undergoing coronary artery bypass grafting (CABG) and who also had paroxysmal AF. Postoperative AF (≤30 days) was recorded at 7% in the botulinum toxin group and 30% in a placebo group, while for AF events beyond this and out to 1 year, the botulinum toxin group had 0% and the placebo group had 27%. The superior performance of the botulinum group was maintained out to three years, with arrhythmia rates at 23.3% compared to 50% in the placebo group [51]. While the neurotoxic effects of the botulinum were anticipated, it was expected to be a short-term benefit and thus the mechanism for the longer-term benefit is not fully understood. The investigators have proposed that a short-term interruption of the autonomic influences on AF may be enough to suppress AF through a mechanism that involves both the prolongation of atrial refractoriness and inhibition of triggering of the PVs. This mechanism is supported by pre-clinical work which showed that temporary inhibition of the GP activity by botulinum toxin was enough to suppress AF for long periods [52]. 

Another interesting data set relates to heart transplant patients, where it was observed that these patients had exceptionally low rates for AF (4.6%) compared to, for example, patients with double-lung transplants (18.9%) [53]. It was hypothesized that the double-lung transplant patients undergo the physical equivalent of a PVI, whereas the heart transplant patients additionally undergo denervation that is somewhat equivalent to GP ablation. A study from the Mayo Clinic had similar findings, showing lower AF rates after heart transplant compared to surgical maze procedures (roughly equivalent to PVI alone) [54].

## 5. Pulsed Field Ablation of Ganglionated Plexi—Pre-Clinical Studies

**Figure 2:**
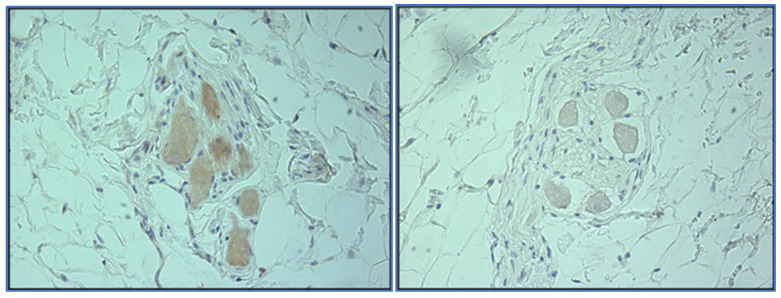
Tyrosine kinase staining of an unablated GP (**left**) and a pulsed field ablated GP (**right**). The unablated control shows uptake of the stain, confirming neuron functionality, while the ablated sample shows no uptake, indicating cell death.

The observation around cardiac denervation in transplant patients and the potential opportunity in terms of an AF treatment led the Mayo Clinic team to explore concepts that could selectively ablate the GPs with minimal damage to the myocardium. This early work initially assessed different energy sources, including RF, ultrasound and DC pulsed electric fields in an open-chest surgical setting, using a canine model; GPs were accessed and ablated with each energy source followed by the measurement of pacing-induced AF induction and histology assessments of the tissue damage [55]. The pulsed electric fields provided the best combination of AF-induction resistance with minimal damage to the underlying myocardium. Using early prototypes of the pulsed electric field catheters, this early work then also included a percutaneous study, using sub-xiphoid access into the pericardial space, giving direct contact to the epicardial fat pads/GPs. AF inducibility was measured before and after GP ablations. The results again showed that resistance to AF induction was increased, with histology showing selective ablation of the neuronal structures within the GPs, as shown in Figure 2. 

This study also explored the possibility of using an extension in atrial effective refractory period (AERP) as a surrogate measure for acute confirmation of GP ablation. It is well recognized that local release of autonomic neurotransmitters from the ganglia shortens the refractoriness of atrial tissue, thereby promoting susceptibility to AF through favourable myocardial substrate [41]. Thus, ablation of the GPs should eliminate neurotransmitter release, thereby enabling a recovery/extension of tissue refractoriness. This initial acute study was followed by a similar study but with chronic time-points out to four months [56]. In this instance, access was achieved in all animals using the sub-xiphoid route; each GP site was ablated with pulses of 1000 V amplitude and pulse width of 100 µs. The number of pulses delivered to each GP ranged from 20 to 60. Energy was delivered in a monopolar configuration with a dispersive pad on the lumbar region of the subjects. The DC pulses were ECG-gated so that they were delivered only during the ventricular refractory period of the cardiac cycle, thereby reducing the risk of inducing ventricular fibrillation through inadvertent myocardial stimulation. AERP was measured before and after ablation at a high right atrium location and within the coronary sinus; this showed that GP ablation provided an average acute AERP extension of 80 ms. Interestingly, when measurements were repeated at the four-month timepoint, much of the AERP extension had retracted, though all animals proved to be still highly resistant to pacing-induced arrhythmia. (This observation is similar to the canine study mentioned earlier, where Botox was used for GP modulation; acute AERP extensions had retracted at 3 months, but the animals were still highly resistant to AF induction. The authors proposed that even a temporary suppression of GP activity may be enough to stop the “AF begets AF” cycle.) Extensive histology evaluations were performed at the GP ablation sites, providing further supporting evidence that the pulse electric field provided preferential ablation of the neuronal cell bodies within the GPs while generally sparing the myocardium from damage. Collateral structures, including the oesophagus, also showed no evidence of ablation injury.

While the ability to successfully ablate the GPs using sub-xiphoid access was now demonstrated, the first clinical use of the technology was planned to be in an open-chest surgical setting. In this context, a further acute canine open-chest study was performed as a full system validation before treating patients. A customized cardiac pulsed field generator was used to deliver the 1000 V/100 µs pulses, with between 30 and 60 pulses applied to each GP. All pulses were again ECG-gated and with droplets of saline infused during pulse delivery to enhance distribution of the electric field into the epicardial fat. Additionally, the electrode-tipped catheters were refined to include an insulation feature on the ‘back face’ to assist with directing the field into the epicardial surface while minimizing spread of the field to collateral tissue and structures [57]. In terms of AERP measurements this study additionally assessed the feasibility of using epicardial AERP readings, for compatibility with clinical open chest surgeries; in this instance, temporary pacing wires attached to the left atrial appendage (LAA) proved to be successful for measuring extensions in AERP. Overall, an average acute AERP extension of 19% was obtained after ablation of all GPs. An interesting observation around local electrograms was the transient increase in current between the local atrial and ventricular potentials; this was typically fully recovered within 10 min and may reflect some reversible electroporation effects in the myocardial tissue. Histology analysis did indeed confirm that there was no damage to the myocardium at the ablation sites. Additionally, oesophageal, phrenic nerve and pericardial tissues showed no evidence of ablation injury.

## 6. In Vitro Cellular Studies

The success of using PFA for performing endocardial pulmonary vein isolation (PVI) has, to a large extent, been assigned to how susceptible cardiac myocytes are to ablation through the mechanism of irreversible electroporation relative to other tissues. An electroporation threshold of 375 V/cm for cardiomyocytes [58] has been widely cited; however, the selective ablation of GPs observed in the aforementioned pre-clinical studies is somewhat at odds with the understanding that myocytes have the lowest electroporation threshold. Fundamental cellular electroporation studies were therefore performed to further explore the characteristics of both myocytes and neurons. An initial study assessed neuronal cells and cardiomyocytes suspended in phosphate-buffered saline during electroporation over a range of field strengths and repeat pulses, using the same pulse width and frequency as used in the pre-clinical studies [59]. Based on cell viability assays, the electroporation threshold for both cell types is similar, at approximately 1000 V/cm when at least 30 repeat pulses are applied. Cell death over time, after electroporation, was also explored using propidium iodide (PI) assays in the parameter range being used pre-clinically (1000 V, 100 µs, 60 pulses). For neuronal cells, there was no notable difference in cell death between 0.5 h and 24 h post-treatment, though myocytes showed delayed cell death out to 24 h.

**Figure 3:**
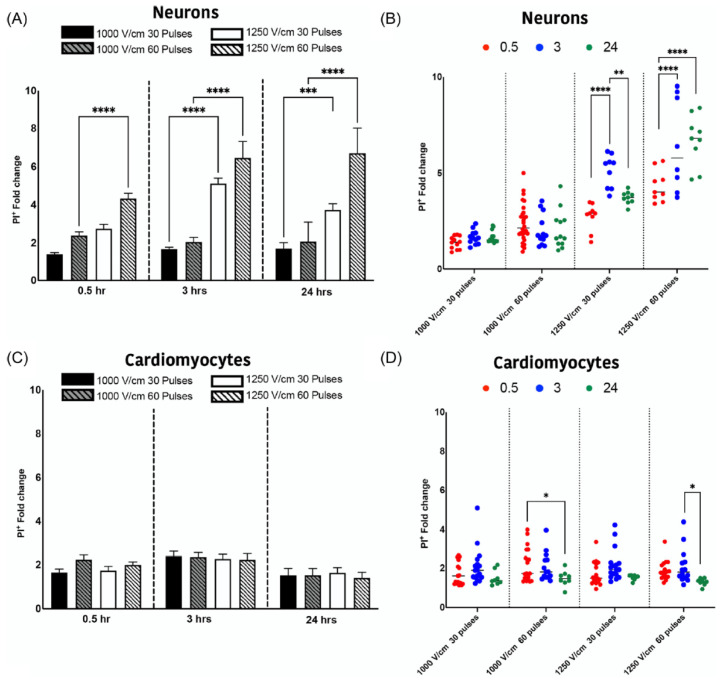
Temporal dynamics of cell death in neurons and cardiomyocytes. The permeability of cells to PI was measured at 1000 V/cm 30 pulses, 1000 V/cm 60 pulses, 1250 V/cm 30 pulses, and 1250 V/cm 60 pulses in neurons (**A**) and cardiomyocytes (**C**). The evolution of PI permeable cells was detected at 0.5 (in red), 3 (in blue) and 24 (in green) hours for neurons (**B**) and cardiomyocytes (**D**). All data shown as mean ± SEM. Statistical significance was performed using two-way ANOVA (* *p* < 0.05, ** *p* < 0.005, *** *p* < 0.001, **** *p* < 0.0001). Reprinted with permission from [60] through the Creative Commons attribution license.

With the desire for a more representative in vitro experiment, a further study was performed, whereby the cells were cultured in a confluent two-dimensional arrangement and then also electroporated in this configuration [60]. Propidium iodide staining showed that at higher field strengths (1250 V/cm), representative of those at the tissue surface during ablation, neurons were more susceptible to electroporation than cardiomyocytes. Additionally, the temporal effects after ablation were noted to be different; neurons showed ongoing, delayed cell death out to 24 h, while cardiomyocytes were more stable, though trending to a small amount of recovery (Figure 3). These temporal differences also probably contribute to the observations of selective ablation of the GPs in the pre-clinical studies, but given the sensitivity to electrical field strength, it is likely that close proximity to the GPs (as obtained with epicardial access) is equally important. 

This study also included a comparison of monophasic and biphasic pulses, using equivalent pulse widths and inter-pulse intervals. Interestingly, for both neurons and cardiomyocytes, the biphasic pulses typically resulted in reduced cell death compared to monophasic, despite the overall range of electrical field in biphasic (±1000 V/cm) being double that of the monophasic (+1000 V/cm). This observation is similar to that reported by others, where there can be a ’cancellation effect’ between the positive and negative phases of biphasic pulses, resulting in higher field strengths being required to achieve the same level of cell death [61]. Additionally, for neurons, shortening of the inter-pulse interval reduced the extent of cell death, while for cardiomyocytes the interval had less of an effect. The inter-pulse interval of 1 s gave the best balance of maximizing neuron cell death and minimizing cardiomyocyte death; this could be a further aspect of the selectivity identified during the pre-clinical studies, where one electrical pulse is delivered per heartbeat.

When considering the differences in electroporation threshold from these recent studies and the widely cited value of 375 V/cm for cardiomyocytes, it is important to note that the 375 V/cm value relates specifically to immature rodent cells (H9C2 myoblasts) that are not necessarily a good representation of mature human cardiac myocytes. Immature cells are more likely to have a lower electroporation threshold. Additionally, quoting the field strength on its own is not particularly informative as pulse width, inter-pulse interval and number of pulses are all part contributing factors in the ablative effect.

Another conundrum with the current data and clinical observations relates to how the phrenic nerve is generally resistant to permanent damage by pulsed electric fields, as described earlier. This may be seemingly at odds with the relative ease at which GPs have been ablated in these studies; however, while both phrenic nerves and GPs are comprised of neuronal cells, it begs consideration that they are very different in structure. The GPs consist primarily of nucleated cell bodies with a relatively large spherical/globular shape located on the epicardial surface of the heart. By contrast, the phrenic nerve is primarily comprised of bundles of long, small diameter axons with nucleated cell bodies located remotely in the brainstem and spinal cord. Given that fundamental models of electroporation indicate larger diameter cells [62], and those with the presence of a larger nucleus [63] have a lower threshold for cell death, the experimental observations, pre-clinically and clinically, both seem reasonable. Additionally the phrenic nerve axons are myelinated, and this is likely to have a protective/insulative effect against electric fields. These factors may explain the high threshold of 3800 V/cm observed when electroporation was applied directly to sciatic nerves (a motor neuron similar to the phrenic nerve) in pre-clinical studies [64]. Separately, it has been reported from electroporation gene transfection studies that nucleated neuronal cell bodies are amongst the most difficult to work with due to their poor ability to recover after permeabilization [65].

## 7. Electric Field Models

**Figure 4:**
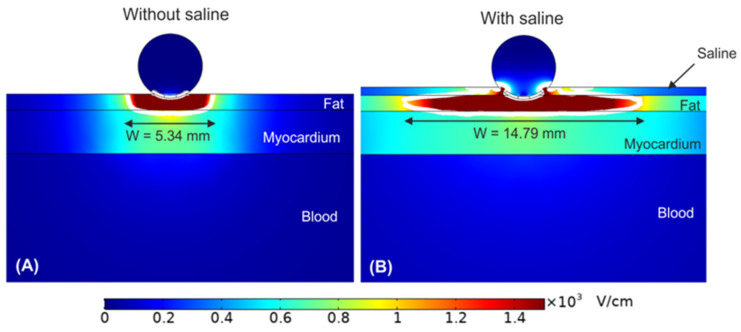
Electric field distribution around the target epicardial site, without (**A**) and with (**B**) a saline layer. The electrode is embedded 0.25 mm in the fat layer. The white contour corresponds to the 1000 V/cm electric field isoline. Reprinted with permission from [66] through the Creative Commons Attribution License.

In order to fully connect the in vitro observations with the pre-clinical findings, two-dimensional electric field models were developed to represent the ablation electrodes in contact with the tissue layers. This epicardial ablation model captured the fat (within which the GPs are embedded), the atrial wall and blood within the atrium. Additionally, the electrode model included the back face electrical insulation as well as saline flow through the electrode, and a dispersive pad to simulate the monopolar arrangement [66]. Pulses of 1000 V amplitude and 100 µs width were applied to the model with the resulting electric field strengths and current flows calculated. There was a number of key findings, but most notable was how the peak electric field was concentrated primarily within the epicardial fat layer, with a sudden drop in field strength at the interface between the fat and myocardial tissue. In principle this would seem to be advantageous as the GPs, embedded within the fat, should be exposed to very high field strengths while the underlying myocardium experiences much lower values. It is, therefore, most likely that this epicardial positioning of the electrodes/catheter is a key factor in the tissue selectivity observed pre-clinically. Another interesting observation of the model was the enhancing effect of saline droplet infusion during ablation. In addition to ensuring electric field conduction into the fat, the saline helped to disperse that field laterally, thereby increasing the volume of fat exposed to the high field strengths. Figure 4 illustrates both of these key findings; this shows how the higher field strengths are retained within the fat and shows the benefit of a thin saline layer on top of the fat surface—the saline increases the lateral spread from 5.34 mm to 14.79 mm. An isoline of 1000 V/cm is shown in white on the electric field distribution. Based on the in vitro cellular data, this value can be viewed as an approximate cut-off for ablation [60]; below this value (outside the isoline), there is likely to be minimal ablation, while above this value (inside the isoline), significant ablation can be expected. It can be clearly seen that the fat is fully retained by this isoline, while the myocardium is primarily outside it. The models therefore support the selectivity observations from the pre-clinical work.

This study also showed how the treatment has a relatively low sensitivity to variable fat thickness. Considering a 1000 V pulse with very thin layers of fat (<0.25 mm), it is inevitable that some myocardium will be exposed to high electric fields (>1000 V/cm). On the other hand, thicker layers of fat are more likely to be present, and even at 5 mm of epicardial fat, electric fields of over 1000 V/cm are still obtained through this full thickness. With fat thickness above 5 mm, higher voltages are needed to ensure full exposure of the fat to high electric fields.

Three-dimensional models were also developed using both a full patient torso as well as a limited-domain model with only a smaller region of interest around the ablation site [67]. The study showed general equivalency between the two approaches, since the electrical field is mainly confined to the target site (epicardial fat); this enabled multiple conditions to be run of the limited-domain model, rather than needing the computationally demanding full torso model each time. It is also re-assuring to see that the average current response generated from the models (6.7 A) was similar to the average currents recorded in the preclinical studies (7.6 A). Importantly, this study also demonstrated that the electrical field transmitted to adjacent organs was very low—less than 30 V/cm at the oesophagus and less than 36 V/cm at the lungs. These values are orders of magnitude below levels, likely to cause irreversible electroporation in these tissues [68,69]. While the absence of oesophageal damage has been widely reported when using pulsed field for endocardial PVI, it is reassuring that electric field models (and pre-clinical studies) also support this observation for epicardial ablation of the GPs.

A separate modelling study additionally explored the effect that an implanted coronary artery stent may have on the electrical field distribution and the potential for any thermal effects due to the presence of the metallic implant [70]. This is of particular interest given the epicardial aspect of the coronary arteries and the possibility that some AF patients may have a prior implanted stent. The model first simulated applying pulses (1000 V, 100 µs) to an unstented artery within the epicardial fat; the simulation included different thickness of fat between the artery and the ablation electrodes. The presence of the artery did cause some disruption of the electric field patterns with increased field just above and below the artery and decreased fields laterally on both sides of the artery; however, the majority of the fat was still exposed to a field strength of at least 1000 V/cm. The field strength in the blood, within the artery, dropped off significantly to approximately 200 V/cm. The presence of a stent did not notably change the electric field distribution within the fat, but the value within the stent dropped to essentially zero, due to the Faraday cage effect. In relation to temperature effects, the aforementioned small zones of increased field strength in the fat translated to peak temperatures of 47.2 °C and 44.5 °C, with and without a stent, respectively, but only when inter-pulse intervals were reduced to 10 µs and with a worst-case scenario of ablating directly on the stented artery. The inter-pulse interval of approximately one second that is used in pre-clinical and clinical work, with monophasic pulses of 1000 V for 100 µs, causes no temperature increase.

## 8. First-in-Human Clinical Studies

**Figure 5:**
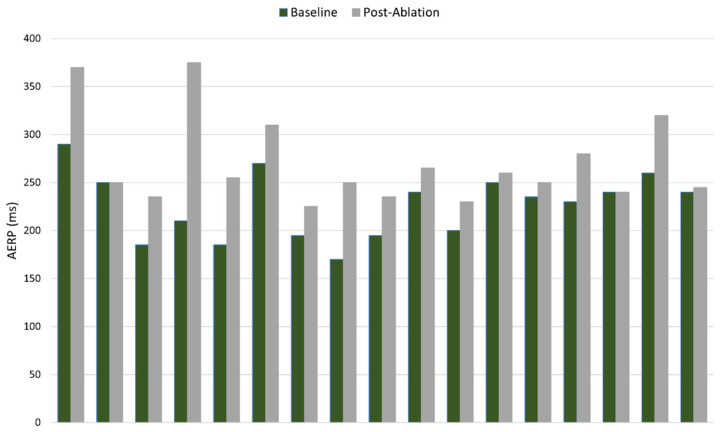
Paired baseline and post-ablation AERP values for patients in safety and feasibility study.

Ultimately, it is expected that targeted GP ablation treatment will be delivered percutaneously using sub-xiphoid access into the pericardial space, to ablate directly on the GPs/fat pads; however, for initial assessment of safety and feasibility, the procedure was performed on patients undergoing elective open-chest coronary artery bypass grafting (CABG) surgery. This first study was an ‘all-comers’ trial, whereby the surgery patients did not need to have a prior history of AF. A total of 24 patients were enrolled in this study (NCT 04775264), and it was performed at the Na Homolce Hospital (Prague, Czech Republic) and Tbilisi Heart & Vascular Clinic (Tbilisi, Georgia) [71]. Immediately after sternotomy, baseline AERP measurements were collected, and each of the target GPs were then ablated (OSGP, TSGP, RSGP, LSGP and LMGP). Ablation parameters were identical to those used pre-clinically: 1000 V monophasic and monopolar, 100 µs pulse width, and 1 pulse of energy given per ECG-gated beat with up to 60 pulses delivered to each GP location. Post-ablation AERP was then measured before the patient progressed to their elective surgery. GP ablation was successful in all patients, and an average acute extension in AERP of 21% was recorded—similar to the pre-clinical studies and promising in terms of the potential to reduce susceptibility to AF. Figure 5 illustrates individual patient baseline and paired post-ablation AERP values. It is noted that there is significant variability in the data—some of this variability is inherently location-dependent for each patient, though it is likely that the measurement method also contributes to this. It remains to be seen if AERP will be a suitable acute measure in real-world clinical settings. Patient follow-ups included clinical assessment and 24 h Holter monitoring at 1, 3, 6 and 12 months. No incidences of AF were recorded during the Holter follow-ups, and no study-related complications occurred. 

Following on from the successful safety and feasibility trial, a second study was performed. Again, this was with patients undergoing elective CABG surgery, but in this instance, a medical history of paroxysmal atrial fibrillation (within the prior 12 months) was a requisite inclusion criteria. A total of 12 patients were enrolled in this study, and it was performed at Tbilisi Heart & Vascular Clinic (NCT05426759). Treatment procedure and follow-ups were identical to the first cohort of patients. Follow-up is currently in progress, but preliminary data show that one patient had a brief (45 min) episode of AF at 3 months that resolved spontaneously. No AF was detected at the 6-month timepoint. 

## 9. Discussion

The involvement of the cardiac autonomic nervous system in AF is now well appreciated; however, its precise contribution and whether it can be modulated or ablated to successfully reduce or eliminate AF remains to be clearly established in clinical settings. The selective ablation of ganglionated plexi (GPs) described here presents the first opportunity to answer this question. The approach is built on a robust scientific background in aspects of cellular physiology, electric field models and pre-clinical work. Understanding is evolving with regard to how the pulsed electric field energy and the epicardial procedure can be optimized to achieve ablation selectivity. 

**Figure 6:**
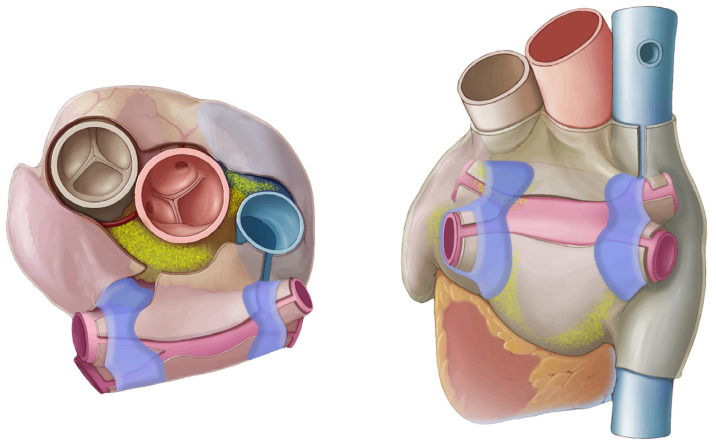
Schematic of endocardial PVI lesions (blue) and epicardial GP locations (yellow).

At this point, the results from pre-clinical work and the early clinical studies are very promising and are reinforced by observations from several other related studies and data sets. The most important of these relates to conventional pulmonary vein isolation and how it is appreciated that collateral injury to some of the GPs may in part contribute to more successful outcomes [72,73]. This is substantiated through studies using high-frequency stimulation (HFS) of GPs, showing abolition of vagal response after PVI; this has been demonstrated for both radiofrequency (RF) and cryoballoon ablation [74,75]. Specifically, the left superior GP is often ablated during isolation of the left superior vein, while the ligament of Marshall GP can be partly ablated during isolation of the left inferior or the left superior pulmonary veins. The inferior left and inferior right GPs are rarely impacted as they are typically too far from the respective inferior pulmonary veins. The transverse sinus GP and right superior GP are also not impacted by PVI; Figure 6 schematically shows this overlap between the PVI lesions and GP location.

A vagal response is often observed during the ablation itself, i.e., induced without HFS, and there is mixed opinion on the significance of this observation, including if abolition of that vagal response is a suitable predictor of a better PVI outcome. Several studies have indeed shown that abolition of the response—and transection of the GPs—is associated with better outcomes at 12 months [76,77].

**Figure 7:**
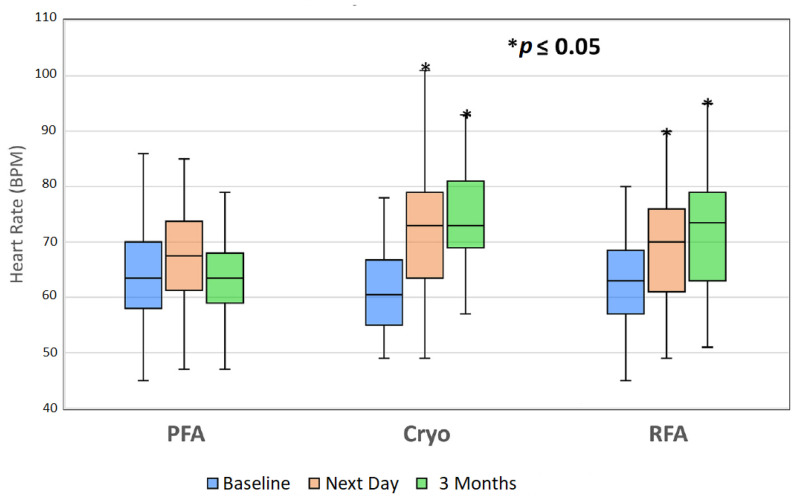
Patient heart rates collected at baseline, day 1 postablation, and 3 months postablation. The asterisk (*) indicates statistical significance (*p* ≤ 0.05, paired *t*-test) compared to baseline heart rates. Total of 40 patients in each group. Bars indicate median heart rate. BPM = beats/min; Cryo = cryoballoon ablation; PFA = pulsed field ablation; RFA = radiofrequency ablation. Reprinted with permission from [78]; Copyright © 2022 Elsevier.

As described earlier, the introduction of endocardial pulsed field PVI is being broadly welcomed, particularly because of the improved safety profile—even if efficacy outcomes appear no better than RF or cryoablation [37]. It is now also widely reported that a vagal response is observed during pulsed field PVI, however, continued/progressive ablation does not eliminate it, and the effect is profound, such that pacing or the administration of atropine is required to manage it [78,79]. Notably, the durable increase in heart rate associated with RF or cryoablation PVI [80] is not observed following endocardial pulsed field PVI; this is shown in Figure 7. This increase in heart rate is attributed to the vagal denervation effects from the collateral GP injury. The absence of this heart rate increase is further evidence that, while GPs may be strongly stimulated, they are not readily ablated during endocardial pulsed field PVI. These autonomic response observations have been supported by studies of biomarkers as surrogates for cardiac and neuronal cell injury. While pulsed field appears to produce higher troponin T release compared to RF or cryoablation (possibly due to larger lesions), thereby indicating substantial myocardial injury [81], pulsed field produces significantly lower S100B release—a marker of neuronal cell injury—compared to cryoballoon ablation [82]. This points to reduced collateral ablation of the GPs by endocardial pulsed field PVI. Overall, these insights have raised some concern that the medium- and longer-term outcomes of pulsed field PVI may ultimately be inferior to RF or cryoablation [83,84,85]. There is possibly a scenario ahead where endocardial pulsed field PVI combined with epicardial GP ablation may provide an optimum outcome in terms of increased ablation success rates.

The significant potential of GP ablation as a stand-alone treatment for AF has also been shown in the GANGLIA-AF study, where GP ablation had a 1-year freedom from AF of 50% compared to 64% for PVI alone, in matched groups of paroxysmal AF patients [86]. Additionally, and most interesting, the GP ablation group had a significantly lower use of anti-arhythmic drugs at 12 months compared to the PVI group. Considering that not all GPs may have been targeted (endocardial HFS mapping potentially not getting the TSGP); this is a promising outcome. Additionally, the use of RF ablation from an endocardial aspect probably caused myocardial ablation, conceivably resulting in an increase in re-entrant atrial tachycardia, potentially confounding the outcome. This group has additionally reported a case where that ablation of a single GP (left inferior) was sufficient to terminate AF in a patient that was having repeated paroxysms at least once a month [87]. In a separate study, a group of 12 patients with vagally mediated AF were successfully treated by ablation of the RSGP alone [88]. Additionally, it is interesting to note that while vagal activation is widely associated with AF, very low levels of vagal stimulation at the tragus nerve may provide protection against AF [89].

GP ablation as a stand-alone procedure is already gaining increased interest for the treatment of vasovagal syncope—the term “Cardioneuroablation” was indeed first coined in this context [90]. Ablation of right-sided GPs is proving to be an attractive alternative to pacemaker implantation [91,92]. GP ablation alone has also been successfully used for the treatment of sinus bradycardia [93]; however, a recent case study highlighted the question around the potential for reinnervation after GP ablation; in this instance, GP ablation was successful acutely, but an implanted monitor provided evidence of a gradual recovery of autonomic innervation and associated return in syncopal events [94]. This reinnervation issue has already been reported in pre-clinical studies of GP ablation for the treatment of AF [95,96]; the main concern is that hyper-innervation after ablation may be proarrhythmic. A key common feature in all of these studies, however, is that RF energy has been used for the ablations. It is increasingly being understood that the thermal effects from this ablation method can indeed stimulate nerve recovery and regrowth through increased plasma levels of nerve growth factor-β (NGF-β) [97,98]. In this context it is noted that the epicardial selective pulsed field ablation of GPs reviewed herein is not reliant on thermal energy for ablation, thereby potentially avoiding promotion of nerve growth factors. From an AF perspective, the GP ablation should therefore, be durable in the long term, with the potential to provide added value to conventional thermal or pulsed field PVI. Where cases of recurrent AF are often associated with fully isolated veins, GP ablation could also address the non-pulmonary vein triggers and residual substrate not treated by PVI [99]. In addition to AF and syncope, neuromodulation is also being explored for treatment of hypertension and heart failure with preserved ejection fraction [100].

Finally, in relation to potential risks associated with GP ablation, concerns have been expressed that it may lead to an imbalance, with excess unopposed sympathetic activity that could lead to ventricular arrhythmias. Much of this insight is coming from ex vivo studies [101] or pre-clinical work, which included periprocedural induction of acute myocardial ischemia [102]. It is difficult to know if this translates to humans, especially given the many studies of GP ablation performed to date with no reports of ventricular arrhythmia. One widely cited clinical case study is somewhat misleading, given the unusual details of the case—the patient went into VF during a ventricular stimulation protocol after PVI (not targeted GP ablation), having developed AF after a leg trauma incident [103]. The authors acknowledged that it was unknown if VF could have been induced before PVI. While the short- and medium-term results of GP ablation are promising, the longer-term safety aspects have not yet been comprehensively evaluated, including any risks associated with partial denervation.

## 10. Future Work

Scientific and clinical evidence collected to date suggest that this epicardial selective GP ablation approach has significant potential in the treatment of AF—either as a concomitant procedure with PVI and/or as a stand-alone treatment in patients with vagally mediated paroxysmal AF. The possibility of higher rates of late AF recurrence with pulsed field PVI may elucidate an unmet need in this regard [104]. Moving forward, there are some specific and broader technology developments that could help with clinical implementation.

### 10.1. Access

As noted, this treatment will be delivered to the epicardial surface of the heart using sub-xiphoid access into the pericardial space. This technique is already used in gaining access for epicardial ablation of ventricular tachycardia, using widely available needle access tools and conventional fluoroscopy techniques [105,106,107]. It has also been used for delivering left atrial appendage ligation devices into position [108]; however, a small subset of clinicians are currently trained in this technique, and it is sometimes perceived to be a difficult and risky procedure. The necessary upskilling is, however, being well facilitated by the on-going development of several novel access tools, which improve safety and significantly streamline the procedure [109,110,111,112,113,114].

### 10.2. Localization

Having gained access, the next step is to identify and navigate to the target location. While high frequency stimulation (HFS) and the resulting vagal response has been used to identify GPs, it has been shown that anatomical localization, using fluoroscopy, is more reliable [115]. While this anatomical technique has been successfully used in many studies, including the pre-clinical work discussed earlier, it does require a high level of knowledge in relation to the epicardial and pericardial anatomy. Adaption of mapping techniques currently used in electrophysiology labs would be desirable. In this context, the use of electroanatomical mapping is already being widely used for GP localization in the treatment of syncope [116,117] and sinus bradycardia [118]. Initial work has also been performed in relation to AF [119,120]. There will be some technical challenges with alignment of these endocardial maps with epicardial anatomy and navigation, but these seem to be resolvable. High-resolution epicardial mapping has already been shown to be feasible for identifying re-entry circuits and foci for ventricular tachycardia [121]; it will be interesting to see if these same approaches can be adapted for atrial mapping of GPs. Nuclear imaging has also been assessed in terms of ability to detect and map GPs; specifically, radionuclide imaging using ^123^I-metaiodobenzylguanidine (mIBG) with single-photon emission computed tomography (SPECT) has been explored and compared to HFS measurements [122]. While this study demonstrated promising feasibility, there is still some work to be done in terms of alignment of SPECT images with CT images of the cardiac anatomy.

### 10.3. Navigation

In the open-chest setting, navigation to the target GP sites is readily performed as demonstrated in the first-in-human study already described. The catheters are designed to be short and relatively stiff with a deflectable tip, controlled from the handle. For the sub-xiphoid access, the catheters will need to be longer so that they can track within the pericardial space (after an anterior access) and low profile to minimize the size of the sub-xiphoid access point. Additionally, a balance of stiffness and flexibility will be required so that the catheter can be pushed to get past any pericardial adhesions but also be able to navigate around the epicardial anatomy and contact the target GP locations. Early prototypes of this design have already been assessed pre-clinically with successful outcomes in terms of navigation [123]. The device is being designed for compatibility with commercially available steerable sheaths to provide additional deflection and steerability. Moving forward with bringing this device into clinical studies, one other expectation is to change the mode of energy delivery from monopolar to bipolar. The monopolar configuration is working successfully in open-chest settings, but the spread to the electric field inevitably leads to some peripheral nerve stimulation in the patient. This can be readily addressed by using paralytics, such as vecuronium or rocuronium, but it would be preferable not to need these. Additionally, the bipolar mode gives more focused and quantifiable energy delivery as it is all retained within the treatment target zone. Pre-clinical work with bipolar energy delivery is currently in progress.

### 10.4. Further Pre-Clinial and Safety Studies

While the safety profile of endocardial pulsed field ablation PVI has been well established at this stage, there are a small number of outstanding aspects to be addressed for the epicardial approach to GP ablation. Foremost amongst these is the risk of coronary artery spasm during energy delivery. It is a relatively rare occurrence and can be addressed by administration of nitroglycerin, but right coronary artery spasm has been reported during endocardial pulsed field ablation at the cavotricuspid isthmus [124]. It is not unique to pulsed field and has also been observed during RF [125] and cryoballoon [126] ablation. Given that the GP ablation is being performed epicardially, there are additional considerations in relation to proximity of the treatment electrodes to the coronary arteries. It is noted that no vasospasm events were observed during the pre-clinical and clinical work already performed, but this will need to be assessed further. Similarly, while concerns around esophageal and phrenic nerve injury have been well addressed for endocardial pulsed field, the closer proximity of the epicardial approach to these structures merits on-going assessment. Of note again, no issues have been detected during the pre-clinical and clinical work already completed, with many of the pre-clinical studies including detailed histological evaluations.

## 11. Future Applications

While the primary focus of the technology is the treatment of AF, there are other potential applications where modulation using epicardial cardioneuroablation may be considered. Vasovagal syncope and sinus bradycardia have already been mentioned. Initial work in these treatments used endocardial RF energy delivery, but as stated previously, there are several advantages to using epicardial selective PFA. These include the minimal collateral myocardial damage and potentially reduced risk of reinnervation. The open-chest work that has already been completed has also highlighted additional scope for the technology in the treatment of postoperative AF (POAF). Depending on the type of cardiac surgery, POAF can occur in 30–50% of patients, bringing additional risk to the patients, delayed discharges and increased costs [127]. Prophylactic selective ablation of GPs during surgery can be performed with little additional operative time and may be a useful approach to reducing or eliminating this problem.

## Data Availability

Not applicable.
